# Mesoscale Modeling of Phase Separation Controlled by Hydrosilylation in Polyhydromethylsiloxane (PHMS)-Containing Blends

**DOI:** 10.3390/nano12183117

**Published:** 2022-09-08

**Authors:** Yao Xiong, Chandan K. Choudhury, Vaibhav Palkar, Raleigh Wunderlich, Rajendra K. Bordia, Olga Kuksenok

**Affiliations:** 1 Department of Materials Science and Engineering, Clemson University, Clemson, SC 29634, USA; 2 Prescience Insilico Pvt. Ltd., Bengaluru 560037, Karnataka, India; 3 Georgia Institute of Technology, Atlanta, GA 30332, USA

**Keywords:** polymer-derived ceramics, phase separation, dissipative particle dynamics, hydrosilylation reaction

## Abstract

Controlling morphology of polysiloxane blends crosslinked by the hydrosilylation reaction followed by pyrolysis constitutes a robust strategy to fabricate polymer-derived ceramics (PDCs) for a number of applications, from water purification to hydrogen storage. Herein, we introduce a dissipative particle dynamics (DPD) approach that captures the phase separation in binary and ternary polymer blends undergoing hydrosilylation. Linear polyhydromethylsiloxane (PHMS) chains are chosen as preceramic precursors and linear vinyl-terminated polydimethylsiloxane (*v*-PDMS) chains constitute the reactive sacrificial component. Hydrosilylation of carbon–carbon unsaturated double bonds results in the formation of carbon–silicon bonds and is widely utilized in the synthesis of organosilicons. We characterize the dynamics of binary PHMS/*v*-PDMS blends undergoing hydrosilylation and ternary blends in which a fraction of the reactive sacrificial component (*v*-PDMS) is replaced with the non-reactive sacrificial component (methyl-terminated PDMS (*m*-PDMS), polyacrylonitrile (PAN), or poly(methyl methacrylate) (PMMA)). Our results clearly demonstrate that the morphology of the sacrificial domains in the nanostructured polymer network formed can be tailored by tunning the composition, chemical nature, and the degree of polymerization of the sacrificial component. We also show that the addition of a non-reactive sacrificial component introduces facile means to control the self-assembly and morphology of these nanostructured materials by varying the fraction, degree of polymerization, or the chemical nature of this component.

## 1. Introduction

Controlling reaction kinetics along with the dynamics of phase separation plays an important role in polymer processing, allowing one to tailor a range of material properties. In particular, controlling the phase separation process in Si-based polymers followed by pyrolysis constitutes a robust strategy to fabricate porous polymer-derived ceramics (PDCs) for a number of applications from water purification [1] and microfiltration [2] to hydrogen storage [3]. The pore size of PDCs fabricated using phase separation can range from a few nanometers [4,5] to several hundred microns [6]. The processing of porous PDCs typically involves blending a sacrificial component and preceramic precursors; crosslinking is typically required prior to the pyrolysis [7,8,9]. Polysiloxanes (PSO) are versatile materials with superior chemical, physical, and electrical properties [10]. Ceramic materials derived from PSO exhibit excellent mechanical strength and high thermal stability while also having relatively low processing temperatures [7,11]. The crosslinking of preceramic polymers prior to pyrolysis prevents the porous structures from collapsing and results in high yields of ceramics [11]. Crosslinking of linear PSO, such as linear polyhydromethylsiloxane (PHMS), results in the formation of a ladder-type network structure [12,13]. The hydrosilylation reaction between the silicon–hydrogen group of PHMS and the vinyl groups of an olefin, such as divinylbenzene (DVB) or vinyl-terminated polydimethylsiloxane (*v*-PDMS), provides a facile method of crosslinking and is widely used in synthesis of organosilicons [14,15,16]. Due to the complete decomposition during pyrolysis, PDMS is often selected as the pore former (or the sacrificial component) to fabricate porous PDCs [9,17,18].

The morphology of phase-separated precursors significantly affects the structure of the PDCs. It had been shown that the obtained macroporous ceramics closely resemble the microstructure of the crosslinked precursors [19]. While the total porosity depends on the fraction of the sacrificial components used [1,2,6], the pore size and pore connectivity of PDCs can be controlled via regulating the dynamics of the phase separation process. For example, choosing a sacrificial component of higher viscosity [20] or a higher fraction of a sacrificial component [2,19,20] results in larger pores. In the system of PHMS crosslinked with *v*-PDMS and a crosslinking enhancer, the size of the generated mesopores was shown to increase with an increase in the molecular weight of *v*-PDMS [21]. The miscibility between the polymeric components directly affects the phase separation and subsequent microstructure of PDCs [18,22]. 

During the hydrosilylation reaction, silicon–hydrogen (Si-H) bond is added across the unsaturated carbon–carbon double bond (C=C) of an olefin, resulting in the formation of the silicon–carbon (Si-C) bond [23]. This reaction takes place in the presence of a catalyst and is often accompanied by various side reactions (such as dehydrogenative silylation, hydrogenation of olefins, isomerization and oligomerization of olefins) [23]. Extensive efforts were directed towards designing efficient and selective catalysts for this reaction; notably, hydrosilylation is regarded as one of the most important industrial applications of homogeneous catalysis [24]. Platinum-based (Pt-based) catalysts are primarily used in various industrial applications [24] and are shown to significantly reduce the side products. For example, a Pt-*N*-heterocyclic carbene (Pt-NHC) catalyst is reported to reduce the side products to a 1% yield, selectively forming primary alkylsilanes [23]. In our DPD framework, we neglect the formation of any side products and only consider the formation of silanes since this reaction is shown to be a dominant reaction of the Pt-catalyzed hydrosilylation [23,24].

Herein, we focus on modeling the phase separation coupled with crosslinking reactions in binary and ternary polymer blends containing linear preceramic precursors (PHMS) and sacrificial linear polymer chains. We characterize the phase separation process hindered by the crosslinking and focus on the effects of chemical nature and degree of polymerization of the sacrificial polymer on the evolution of the characteristic length scale in the system. We use the dissipative particle dynamics (DPD) approach [25,26,27], which is an efficient mesoscale approach utilizing soft repulsion potentials. DPD has been used to model a variety of polymer systems [28,29,30,31,32,33,34,35] and has been adapted to model various reactions within polymer solutions, melts, and polymer networks [36,37,38,39,40,41,42,43,44]. In what follows we first introduce our modeling approach and model parameters. We then utilize this approach to model the phase separation and crosslinking due to the hydrosilylation reaction. Specifically, we first focus on the phase separation in the binary system containing PHMS blended with *v*-PDMS. Prior experiments have shown [21] that *v*-PDMS can serve both as a solvent and as a crosslinker and thereby is effectively acting as templating agent controlling the pore size. We characterize the effects of the degree of polymerization and composition on the phase separation and resulting characteristic length scale in the systems. We then consider ternary systems, in which we introduce a second sacrificial non-reactive component. By considering methyl-terminated PDMS (*m*-PDMS), polyacrylonitrile (PAN), and poly(methyl methacrylate) (PMMA), we probe the effect of the chemical nature of this third non-reactive component on the characteristic features of the resulting morphologies formed. 

## 2. Materials and Methods

### 2.1. Dissipative Particle Dynamics Approach

We first briefly introduce the DPD approach we use to model the phase separation and crosslinking reaction; more details of the DPD methodology can be found in the original publications [25,26,27]. The beads in DPD represent groups of atoms; the motion of these beads is governed by Newton’s equations of motion [27]: $dr_{i}/dt=v_{i}$, $dp_{i}/dt=\underset{j\neq i}{\sum }F_{ij}$, where $r_{i}$, $v_{i}$, and $p_{i}=mv_{i}$ are the position, velocity, and momentum of a particle i of mass m, respectively, and $F_{ij}$ is the pairwise additive force exerted on the particle i by the particle j. The summation is taken over all the particles within the chosen cut-off distance $r_{c}$, which effectively introduces a characteristic length scale of the model. The total force acting between the non-bonded beads consists of three contributions [27]: $F_{ij}=F_{ij}^{C}+F_{ij}^{D}+F_{ij}^{R}$, where $F_{ij}^{C}$ is a purely repulsive conservative force, $F_{ij}^{D}$ is dissipative, and $F_{ij}^{R}$ is a random force. We chose the conservative force based on the soft repulsion potential [27]:

(1) $$F_{ij}^{C}=\left\{\begin{array}{c} a_{ij}\left(1-\frac{r_{ij}}{r_{c}}\right)e_{ij}\\ 0 \end{array}\right.\;\begin{array}{c} \left(r_{ij}<r_{c}\right)\\ \begin{array}{c} \left(r_{ij}\geq r_{c}\right) \end{array} \end{array}$$

where $a_{ij}$ is the maximum repulsion between the beads i and j, $r_{ij}=\left|r_{ij}\right|$ is the distance between the centers of these beads, $r_{ij}=r_{i}-r_{j}$, and $e_{ij}=r_{ij}/r_{ij}$. The repulsion parameter between the beads of the same type, $a_{ii}$, is typically calculated based on the degree of coarse-graining [27,45]. Herein, we chose $a_{ii}=78\;k_{B}T/r_{c}$; this parametrization is derived based on the compressibility of water and corresponds to coarse-graining of three water molecules into a single bead [45], so that the volume of a single bead is approximately $90{\;\text{Å}}^{3}$. The repulsion parameter for the dissimilar beads, $a_{ij}$, is chosen based on the affinity between the moieties these beads represent as detailed below. Remaining contributions to the total force read [27] $F_{ij}^{D}=-\gamma \omega^{D}\left(r_{ij}\right)\left(e_{ij}\cdot v_{ij}\right)e_{ij}$ and $F_{ij}^{R}=\sigma \omega^{R}\left(r_{ij}\right)\zeta_{ij}\Delta t^{-1/2}e_{ij}$, respectively, where γ and σ are the strengths of the dissipative and random forces, $v_{ij}=v_{i}-v_{j}$ is the relative bead velocity, and Δt is the simulation time step. The $\zeta_{ij}$ is a symmetric Gaussian distributed random variable with zero mean and unit variance, $<\zeta_{ij}\left(t\right)>=0,\langle \zeta_{ij}\left(t\right)\zeta_{ij}\left(t^{\prime }\right)\rangle =(\delta_{ik}\delta_{jl}+\delta_{il}\delta_{jk})\delta \left(t-t^{\prime }\right)$. The $F_{ij}^{D}$ and $F_{ij}^{R}$ are coupled through the fluctuation-dissipation theorem [26] for which the following two conditions are imposed [27]:$\;\omega^{D}\left(r_{ij}\right)={\left(\omega^{R}\left(r_{ij}\right)\right)}^{2}$, and $\sigma^{2}=2\gamma k_{B}T/m$, where $k_{B}$ is the Boltzmann constant and T is temperature. We chose the weight function [27] $\omega^{R}\left(r_{ij}\right)=\left(1-r_{ij}/r_{c}\right)$ for $r_{ij}\leq r_{c}$. Within the polymer strands, the beads are connected by harmonic bonds with the interaction potential $U_{bond}=K_{bond}{\left(r_{ij}-r_{b}\right)}^{2}/2$, where $K_{bond}=10^{3}\;k_{B}T/r_{c}^{2}$ and $r_{b}=0.7\;r_{c}$ are chosen [46] as the spring constant and the equilibrium bond length, respectively.

To minimize unphysical topological crossings of bonded polymer chains (a known limitation of DPD), we adapted the modified Segmental Repulsive Potential (mSRP) [47,48,49] formulation. Notably, the mSRP DPD formulation captures the effects of entanglements in polymer melts [47]. Within the mSRP DPD framework, pseudo beads are introduced at the center of each bond for all the bonds initially present in the system; the pseudo beads interact with other pseudo beads with a soft repulsion potential defined as [47]:

(2) $$F_{ij}^{SRP}=\left\{\begin{array}{c} b\left(1-d_{ij}/d_{c}\right)e_{ij}^{S}\\ 0 \end{array}\right.\;\begin{array}{c} \left(d_{ij}<d_{c}\right)\\ \begin{array}{c} \left(d_{ij}\geq r_{c}\right) \end{array} \end{array}$$

where b defines the strength of the interaction between the pseudo beads, $d_{ij}=\left|d_{ij}\right|$ is the distance between these beads located at the centres of the bonds, $e_{ij}^{S}=d_{ij}/d_{ij}$, and $d_{c}$ is the cut-off distance for the mSRP interactions. Herein, the mSRP framework is modified [48] to create additional pseudo beads for the bonds created during crosslinking. For the mSRP potential, we set [47] $b=80\;k_{B}T/r_{c}^{2}$ and $d_{c}=0.8\;r_{c}$; these parameters are shown to effectively minimize the bond crossings [47,49]. The strength of the random force is chosen as σ=3, the number density in simulations is $\rho =3/r_{c}{}^{3}$, and the time step is Δt=0.02 τ, where τ is the reduced unit of time [27]. The $r_{c}$, temperature, and the mass of a DPD bead are set at 1.0 in reduced DPD units [27,45]. With the above choice of the degree of coarse-graining, the dimensionless units of length and time correspond to [45] $r_{c}\;\approx$ 0.65 nm and τ≈ 88 ps, respectively. All the results below are provided in the reduced DPD units. PHMS is represented by a linear chain that consists of 30 DPD beads ($N_{\mathrm{PHMS}}=30$) and we vary the number of beads comprising the *v*-PDMS chains, $N_{v-\mathrm{PDMS}}$. The size of the simulation box is L×L×L, where L=40 in reduced DPD units as provided above, periodic boundary conditions are applied in all directions. With the chosen density of 3, the total number of beads in the system is $1.92\times 10^{5}$. Prior to the production runs, the blends are equilibrated for $6\times 10^{6}$ simulation timesteps with the same value of the repulsion coefficient ($a_{ij}=78$) chosen between all the beads within each system; no reactions occur during the equilibration. At the initial timestep of the production run, the repulsion parameters are modified between the beads using the values of $a_{ij}$ provided below. The equations of motion are integrated using the DPD module as implemented in the LAMMPS simulation package [50,51] with mSRP code [47] and the most recent modification of the mSRP code allowing one to use mSRP approach simultaneously with breaking or creating polymer bonds [48,49]. All simulation snapshots were visualized using the Visual Molecular Dynamics (VMD) software [52], and Wolfram Mathematica software [53] was used to generate all the plots.

### 2.2. Defining Repulsion Parameters $a_{ij}$ between Dissimilar Beads

The repulsion parameter for the dissimilar beads is chosen based on the affinity between the moieties these beads represent as [27] $a_{ij}=a_{ii}+3.27\chi_{ij}$ for the chosen density, where $\chi_{ij}$ is the Flory-Huggins interaction parameter. The values of $\chi_{ij}$ can be estimated using the respective solubility parameters $\delta_{i}$ and $\delta_{j}$ as $\chi_{ij}=V_{s}{\left(\delta_{i}-\delta_{j}\right)}^{2}/RT$, where $V_{s}$ is the molar volume of solvent and R is the gas constant. Based on direct comparison with a number of experimental studies, a modified empirical expression for $\chi_{ij}$ is often found to be more reliable for polymer solutions [54,55]: $\chi_{ij}=\beta +V_{s}{\left(\delta_{i}-\delta_{j}\right)}^{2}/RT$ The value of the empirical constant β is typically taken as [54,55] β=0.34, specifically in cases when calculated values of $\chi_{ij}$ (without β correction) are below 0.3. The solubility parameters used in this study are taken from the literature where possible and are provided in Table S1 of Supplementary Materials. The solubility parameter of PHMS was estimated using the group molar attraction constants, $F^{z}$, as [55,56,57] $\delta =\rho \underset{z}{\sum }F^{z}/M$, where ρ is the density, M is the molar weight of the repeat unit, and the summation is taken over all groups of the repeat unit. The relevant values of $F^{z}$ are listed in Table S2 of Supplementary Materials. The same repulsion parameters are used for all the beads within each polymer chain, it is also assumed that changing a bead state from reactive to non-reactive does not affect the beads’ affinity. Finally, the values of $\chi_{ij}$ calculated based on the solubility coefficients and the respective repulsion coefficients $a_{ij}$ are provided in Tables S3 and S4 of Supplementary Materials, respectively. We take the temperature T=298.15 K at which the reference polymer system of interest in this study was crosslinked in the prior experiments [21]. Notably, with the estimated value of $a_{\mathrm{PDMS}-\mathrm{PHMS}}=79.12$, single beads of PHMS are miscible with PDMS, while polymer chains of PHMS and PDMS of the chosen length are immiscible. Hence this choice of the interaction parameter is consistent with experimental observations, showing both initial compatibility between the PDMS and PHMS molecules, and the phase separation between these moieties for sufficiently high molecular weight of PHMS [19,58].

### 2.3. Introducing Hydrosilylation Reaction within DPD Framework

**Figure 1:**
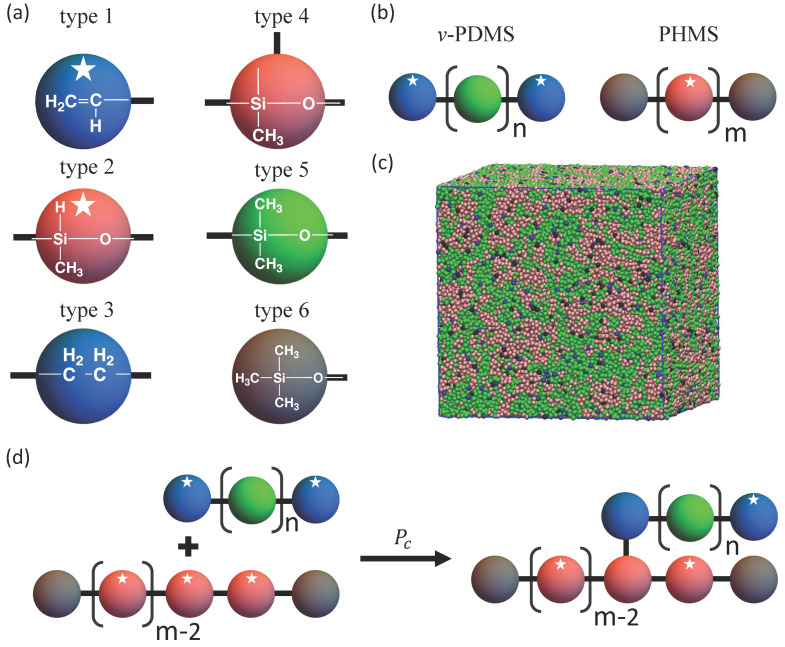
(**a**) Types of coarse-grained beads, “★” indicates that the bead is reactive, (**b**) schematic representation of the *v*-PDMS and PHMS chains, (**c**) schematic of the hydrosilylation reaction, (**d**) initial morphology snapshot (generated with $a_{ii}=78$ for all the beads; no reactions occur during the equilibration) for a sample with $f_{v-\mathrm{PDMS}}=0.5$, $N_{\mathrm{PHMS}}=30$, and $N_{v-\mathrm{PDMS}}=100$. The interaction parameters between all the types of beads are provided in Table S4 of Supplementary Materials.

We model the crosslinking between PHMS and *v*-PDMS chains due to hydrosilylation [21,58] as a stochastic process, similar to the approach previously used within the DPD framework to model polymerization of linear chains [36,38] and various reactions within polymer networks [37,39,40]. The mapping of the group of atoms within the polymer chains into the coarse-grained DPD beads is shown in Figure 1a. In this study, all initial components are linear chains (Figure 1b) composed of $N_{X}$ beads, where X indicates the type of chemical species. Correspondingly, $N_{X}$ represents the degree of polymerization of the individual chains prior to crosslinking. For example, a PHMS chain with $N_{\mathrm{PHMS}}=30$ consists of 28 beads of type 2 and two beads of type 6 (Figure 1a,b). The end vinyl group of *v*-PDMS and the repeat unit of PHMS are represented by a single DPD bead each, as shown in Figure 1a. The effective reactivity of these beads is set by selecting their states (reactive or non-reactive). The reactive moieties are classified as type 1 and type 2 beads for *v*-PDMS and PHMS, respectively (Figure 1a). Once a bond forms between the type 1 and type 2 beads, they become non-reactive and their type is changed to type 3 and type 4, respectively (Figure 1d). For simplicity of representation, we do not highlight the state of reactive beads in the snapshots below. The crosslinking reaction is attempted at each reaction time step $\tau_{r}$, which is ten times larger than the DPD time step ($\tau_{r}=10\Delta t$), same as in previous DPD simulations of reactive systems [36,37,38,39,40].

During each reaction step, for each reactive bead all complementary reactive beads that are within the reaction interaction distance [36,37,38,39,40], $r_{int}=0.7$, are identified as possible bond partners. Further, within this list of possible bond partners, the closest bead is identified as the sole bond partner for the particular bead. The reaction probability is only evaluated for the pairs in which both beads are identified as respective sole partners. A random number between 0 and 1 is generated; the bond is formed if this number is smaller than the chosen probability of reaction, $P_{c}$. Each successful reaction results in an irreversible bond formation, with the bond energy given by $U_{bond}$ defined above; the state of the two beads involved in the crosslinking is correspondingly changed to non-reactive. We assume that changing the bead state from reactive to non-reactive does not affect the affinity of this bead. We set $P_{c}=$ $10^{-3}$ as the reference value of the reaction probability. As we show below, this choice of the reference value of $P_{c}$ results in the first order reaction kinetics of the consumption of the vinyl groups provided that there is an abundant number of PHMS with respect to the vinyl groups in the system. Notably, prior experimental studies showed the consumption of silicon–hydrogen groups to obey first-order kinetics [59,60,61]. In experiments, an increase in the catalyst concentration or reaction temperature speeds up the hydrosilylation reactions [61]. Finally, prior to all the production runs in this study, the systems are equilibrated as described above; an example of an equilibrated system with $f_{v-\mathrm{PDMS}}=0.5$, $N_{\mathrm{PHMS}}=30$, and $N_{v-\mathrm{PDMS}}=100$ is shown in Figure 1c.

## 3. Results

### 3.1. Hydrosilylation Reaction Arrests Domain Growth in Blends Containing PHMS

**Figure 2:**
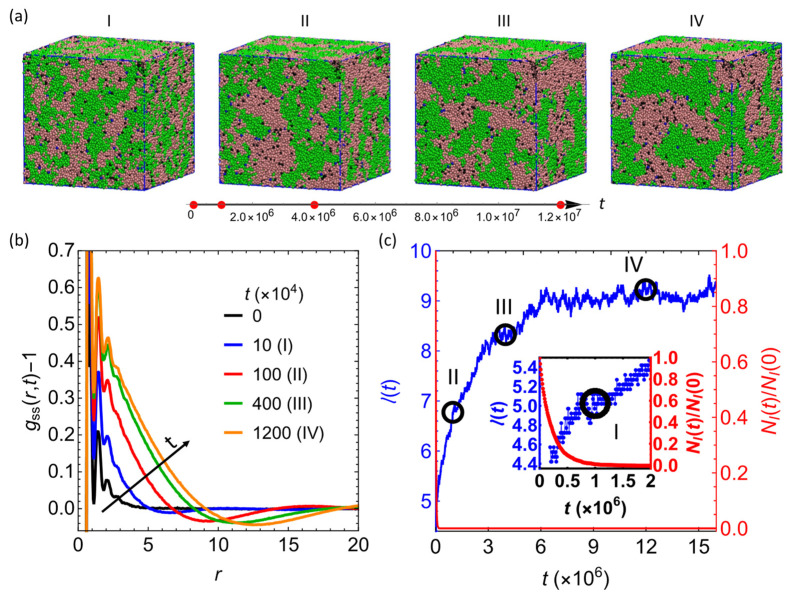
(**a**) Snapshots (I–IV) of the blend with $f_{v-\mathrm{PDMS}}=0.5$ and $N_{v-\mathrm{PDMS}}=100$. The time steps from the left to the right are $1\times 10^{5}$, $1\times 10^{6}$, $4\times 10^{6}$, and $1.2\times 10^{7}$. (**b**) The $g_{ss}\left(r,t\right)-1$ at various times as listed in the legend. (**c**) The time evolution of the characteristic length scale, $l\left(t\right)$, and the number of vinyl groups in the system normalized by the initial number of these groups, $N_{I}\left(t\right)/N_{I}\left(0\right)$. The time instances corresponding to the snapshots in (**a**) are marked by the open circles. The inset shows evolution at early times.

In the first series of simulations, we characterized the time evolution of the 50/50 reactive blend (the fraction of *v*-PDMS, $f_{v-\mathrm{PDMS}}=0.5)$ containing 3200 PHMS chains with the degree of polymerization $N_{\mathrm{PHMS}}=30$ and 960 *v*-PDMS chains with $N_{v-\mathrm{PDMS}}=100$. Representative simulation snapshots are shown in Figure 2a; the time steps corresponding to these snapshots are marked by the red dots on the arrow below the images. Initially, the chains are well intermixed and equilibrated as detailed in Materials and Methods section. The phase separation between the PHMS and *v*-PDMS chains results in the aggregation of the chains of the same type with time as evident in these snapshots and in the corresponding increase in the characteristic domain size quantified below. The sacrificial phase is shown in green (bead type 5) and blue (bead types 1 and 3), and the matrix phase (PHMS chains) is shown in dark pink (bead types 2 and 4) and black (bead type 6), respectively (please refer to Figure 1a for definition of bead type). Note that in the absence of the hydrosilylation reaction, macroscopic phase separation is observed in the same system with two large domains forming upon equilibration (Figure S1a in Supplementary Materials). The hydrosilylation reaction reduces the mobility of polymer chains and arrests the macroscopic phase separation, correspondingly limiting the characteristic domain size.

To quantitatively characterize evolution in this system, we track the time evolution of the characteristic size within the system, $l\left(t\right)$, and a fraction of vinyl groups that remain reactive (beads of type 1 in the scheme given in Figure 1a) with respect to the initial number of the vinyl groups available. In the multi-component system, it is convenient to use partial radial distribution functions (RDFs) $g_{\alpha \beta \;}\left(r,t\right)$ to assess the structural properties during the evolution and upon reaching an equilibrium, where the indexes α and β refer to the types of beads in the multicomponent system. The $g_{\alpha \beta \;}\left(r,t\right)$ for the beads of types α and β is computed via the beads’ coordinates as [62]:

(3) $$c_{\alpha \;}c_{\beta \;}g_{\alpha \beta \;}\left(r,t\right)=\frac{1}{\rho }\langle \frac{1}{N}\;\overset{N_{\alpha }}{\underset{i=1}{\sum }}\overset{N_{\beta }}{\underset{j\neq i}{\sum }}\delta (r\left(t\right)+r_{i}\left(t\right)-r_{j}\left(t\right))\rangle$$

where ρ is the total number density of the system of N beads; $c_{\alpha ,\beta }=N_{\alpha ,\beta }/N$ denotes the fractions of beads of types α and β in the multicomponent system, $r_{i,j}$ are the coordinates of beads α and β, respectively; $\delta \left(\ldots \right)$ is the Dirac δ-function, and 〈…〉 denotes an ensemble average at the given time instant t. It is instructive to group all the *v*-PDMS beads in our systems (bead types 1, 3, and 5), and all the beads comprising PHMS chains (bead types 2, 4 and 6). We will refer to these groups of beads as sacrificial (denoted by the subscript “s”) and as matrix beads (denoted by the subscript “m”), respectively. To characterize the domains formed by the sacrificial beads, we track the time evolution of the partial RDFs of all the sacrificial beads $g_{ss\;}\left(r,t\right)$ in the simulations below. Notably, the total RDF in this system can be calculated as $g\left(r\right)=c_{ss\;}^{2}g_{ss\;}\left(r,t\right)+2c_{ss\;}c_{mm\;}g_{sm\;}\left(r,t\right)+c_{mm\;}^{2}g_{mm\;}\left(r,t\right)$, where $g_{mm\;}\left(r,t\right)$ is the partial RDF for all the beads comprising PHMS chains.

The shifted partial RDFs of the sacrificial component, $g_{ss}\left(r,t\right)-1$, at the time instants corresponding to the morphologies in the snapshots in Figure 2a, are plotted in Figure 2b. All $g_{ss}\left(r,t\right)-1$ curves exhibit primary peaks at the separation r≈0.7 corresponding to the bond length between the beads of the polymer chains set by the choice of the harmonic bond potential (see Materials and Methods section above). There are also additional smaller amplitude oscillations in $g_{ss}\left(r,t\right)$ at relatively short distances, which can be attributed to the packing of the nearest-neighbor and next-nearest-neighbor [63] beads. At all distances beyond this small distance, $g_{ss}\left(r,t\right)-1$ attains approximately zero values at initial times (t=0, solid black line in Figure 2b), indicating that the sacrificial beads are essentially uniformly distributed at these length scales.

The magnitude of $g_{ss}\left(r,t\right)-1$ increases with time until the system reaches an equilibrium, confirming the aggregation of the sacrificial beads into distinct domains during the phase separation arrested by the hydrosilylation reaction. The characteristic length scale within this system can be defined in a straightforward manner via zero crossing [64,65,66] of $g_{ss}\left(r,t\right)-1$. Specifically, herein we quantify the characteristic length scale, $l\left(t\right)$, via first value of r at which the function $g_{ss}\left(r,t\right)-1$ crosses zero from a positive to a negative value (Figure 2b). Alternatively, the characteristic length scale can also be calculated from the first minimum [66] of $g_{ss}\left(r,t\right)$ at distances exceeding the short distance correlations discussed above. It had been previously shown that the characteristic length scales calculated from zero crossings of $g_{\alpha \alpha }\left(r,t\right)-1$ obey expected scaling during spinodal decomposition [64,66]. Further, it had also been shown that the ratio between the characteristic length scales corresponding to the minima of RDF and those calculated from the zero crossing of the shifted RDF remains approximately constant in various systems undergoing phase separation [64,66]. This is also the case for the simulation in Figure 2a, as shown in Figure S2 of Supplementary Materials.

The characteristic length scale $l\left(t\right)$ (in blue, left axis) and the number of the reactive vinyl groups (beads of type 1), $N_{I}\left(t\right)$, with respect to the initial number of these beads, $N_{I}\left(0\right)$ (in red, right axis), are given in Figure 2c. The value of $N_{I}\left(0\right)$ is defined by the number of *v*-PDMS chains in the system and can be expressed as:

(4) $$N_{I}\left(0\right)=2f_{v-\mathrm{PDMS}}\rho L^{3}/N_{v-\mathrm{PDMS}}$$

where ρ and L are the dimensionless beads number density and the linear size of the simulation box as defined in Models section. The open circles on this plot mark the time instants that are chosen in the snapshots in Figure 2a, the inset shows the time evolution of both $l\left(t\right)$ and $N_{I}\left(t\right)/N_{I}\left(0\right)$ at early times. These results clearly show that the reactive vinyl beads are consumed significantly faster than the time required for the phase separation process to reach equilibrium and correspondingly for $l\left(t\right)$ to reach its value at saturation, $\bar{l}$. The observed ratio $N_{I}\left(t\right)/N_{I}\left(0\right)$ decays exponentially; however, the apparent reaction rate calculated via exponential fitting is lower due to the contribution from the diffusion processes than that in the case of an abundant number of PHMS (Figure S3a). In the latter case ($f_{v-\mathrm{PDMS}}=0.1$), the consumption of the vinyl groups obeys the first order reaction kinetics with the reaction rate $\approx P_{c}/\tau_{r}$ due to the abundant number of reactive PHMS beads available for each reactive vinal group bead (Figure S3a). Notably, prior experimental studies showed that the consumption of vinyl groups follows the first-order kinetics [59,60,61,67]. The effect of the crosslinking reaction kinetics on the equilibrium morphology is probed by reducing the reaction probability from the reference value $P_{c}=10^{-3}$ to $5\times 10^{-4}$ (Figure S3b,c). The first order reaction kinetics with the reaction rate $P_{c}/\tau_{r}$ in the limit of an abundant number of PHMS ($f_{v-\mathrm{PDMS}}=0.1$) also holds for the lower value of $P_{c}$ probed. The evolution of the characteristic length scale (Figure S3c) at these two probabilities shows that a slower crosslinking reaction results in somewhat faster increase in l at early times, but approximately the same equilibrium domain size is reached (Figure S3c). More significant decrease in $P_{c}$ would result in an increase of the characteristic size, with macro phase separation observed in the limit of zero reaction rates, as shown in Figure S1.

### 3.2. Effect of Degree of Polymerization of the Sacrificial Chains, $N_{v-PDMS}$

**Figure 3:**
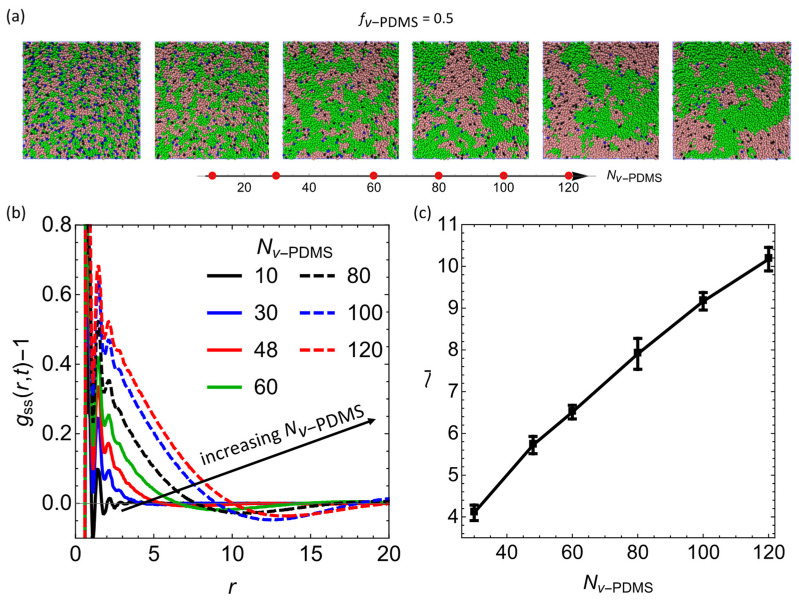
Effect of the degree of polymerization of the sacrificial component, $N_{v-\mathrm{PDMS}}$. (**a**) Equilibrium morphology snapshots of the blends with $f_{v-\mathrm{PDMS}}=0.5$ and various $N_{v-\mathrm{PDMS}}$. The values of $N_{v-\mathrm{PDMS}}$ from the left to the right are 10, 30, 60, 100, and 120. (**b**) The $g_{ss}\left(r\right)-1$ at various $N_{v-\mathrm{PDMS}}$ as listed in the legend. (**c**) The average equilibrium characteristic length scale $\bar{l}$. Here and below, $\bar{l}$ is averaged over the data taken within the last five frames (upon reaching an equilibrium) for four independent simulation runs; the error bars correspond to the standard deviation.

In the next series of simulations, we focus on the effect of the degree of polymerization of *v*-PDMS, $N_{v-\mathrm{PDMS}}$, on the average characteristic size upon reaching an equilibrium, $\bar{l}$. We vary $N_{v-\mathrm{PDMS}}$ within the following range: $N_{v-\mathrm{PDMS}}= [10,\;30,\;48,\;60,\;80,\;100,\;120]$. Representative snapshots upon reaching the equilibrium with selected values of $N_{v-\mathrm{PDMS}}$ are shown in Figure 3a. The shifted partial RDFs, $g_{ss}\left(r\right)-1$, corresponding to the snapshots in Figure 3a are provided in Figure 3b for the same selected values of $N_{v-\mathrm{PDMS}}$. This plot shows that at the lowest degree of polymerization of the sacrificial component considered, $N_{v-\mathrm{PDMS}}=10$, $g_{ss}\left(r,t\right)-1$ remains approximately zero beyond the initial small distances, indicating that the blend remains well intermixed on these length scales. An increase in $N_{v-\mathrm{PDMS}}$ leads to the distinct increase in $\bar{l}$. To understand this effect, recall that at a constant fraction of the sacrificial components ($f_{v-\mathrm{PDMS}}=0.5$ in this series of simulations), higher values of $N_{v-\mathrm{PDMS}}$ correspond to a smaller number of chains and, respectively, to a lower number of reactive vinyl groups available, $N_{I}\left(0\right)$ Equation (4). Hence, an increase in $N_{v-\mathrm{PDMS}}$ results in a decrease in the number of crosslinks formed in the system upon equilibration, in turn providing less constraints on the phase separation and resulting in formation of larger sacrificial domains. This tendency is clearly evident from the shift of zero crossing of $g_{ss}\left(r\right)-1$ to the higher values of r for larger $N_{v-\mathrm{PDMS}}$ (Figure 3b).

To characterize the average equilibrium length scale of the sacrificial domains, $\bar{l}$, herein and in the subsequent series of simulations, we output the RDF data every $5\times 10^{4}$ time steps and average the value of l calculated within the last five frames (upon reaching an equilibrium) for four independent simulation runs. An increase in l with an increase in $N_{v-\mathrm{PDMS}}$ is shown in Figure 3c, with error bars corresponding to standard deviation. Hence our mesoscale simulations demonstrate formation of larger sacrificial domains with an increase in $N_{v-\mathrm{PDMS}}$; similar trend showing an increase in the pore sizes with an increase in the molecular weight of *v*-PDMS was observed in a previous experimental study [21].

### 3.3. Effect of the Fraction of the Sacrificial Component, $f_{v-PDMS}$

**Figure 4:**
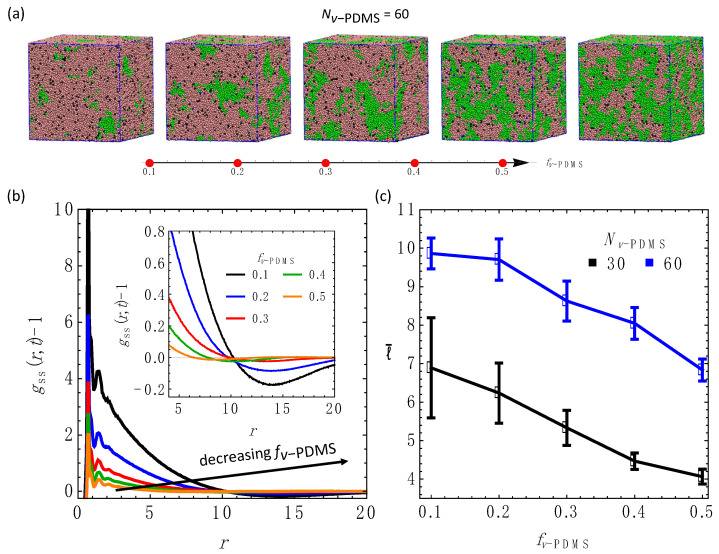
Effects of fraction of sacrificial component, $f_{v-\mathrm{PDMS}}$. (**a**) Snapshots of the blends with $N_{v-\mathrm{PDMS}}=60$ and various $f_{v-\mathrm{PDMS}}$. The values of $f_{v-\mathrm{PDMS}}$ from the left to the right are 0.1, 0.2, 0.3, 0.4, and 0.5. (**b**) The $g_{ss}\left(r\right)-1$ at various $f_{v-\mathrm{PDMS}}$ as listed in the legend. (**c**) The average equilibrium characteristic length scale $\bar{l}$ as a function of $f_{v-\mathrm{PDMS}}$ for $N_{v-\mathrm{PDMS}}=30$ (in black) and $N_{v-\mathrm{PDMS}}=60$ (in blue).

In the next series of simulations, we focus on the effect of the volume fraction of the sacrificial component, $f_{v-\mathrm{PDMS}}$, on the equilibrium morphology. We vary $f_{v-\mathrm{PDMS}}$ as following: [0.1, 0.2, 0.3, 0.4, 0.5]. Equilibrium snapshots of the systems with $N_{v-\mathrm{PDMS}}=60$ and the above values of $f_{v-\mathrm{PDMS}}$ are shown in Figure 4a. The corresponding snapshots of the sacrificial domains only are provided in Figure S4, where beads belonging to the distinct clusters are marked using the same color. These snapshots show that the sacrificial beads form large, interconnected clusters (Figure S4). A decrease in $f_{v-\mathrm{PDMS}}$ results in a clear shift of the $g_{ss}\left(r\right)-1$ zero crossing towards higher values of r (Figure 4b), corresponding to the formation of larger sacrificial domains. Secondary shallow peaks in $g_{ss}\left(r\right)$ are distinguishable at lower values of $f_{v-\mathrm{PDMS}}$ (inset in Figure 4b), indicating an average distance between the smaller domains. The characteristic average length scale in equilibrium, $\bar{l}$, decreases with an increase in $f_{v-\mathrm{PDMS}}$ for both values of $N_{v-\mathrm{PDMS}}$ probed in these series of simulations (data corresponding to $N_{v-\mathrm{PDMS}}=30$ and $N_{v-\mathrm{PDMS}}=60$ are shown in black and blue, respectively). This decrease is most pronounced for the highest $f_{v-\mathrm{PDMS}}$, while the value of $\bar{l}$ approximately saturates at lower $f_{v-\mathrm{PDMS}}$. The vinyl groups in all the simulations are completely consumed via the crosslinking reactions, resulting in a smaller number of crosslinks in systems with lower $f_{v-\mathrm{PDMS}}$. Hence, larger sacrificial domains are formed in the systems with the smaller number of crosslinks (Figure 4c).

### 3.4. Ternary Systems Incorporating Reactive and Non-Reactive Sacrificial Components

**Figure 5:**
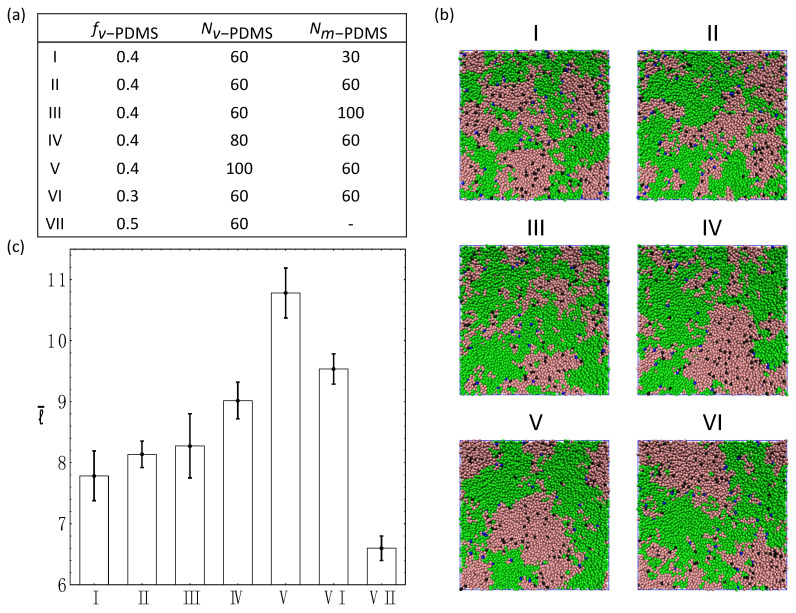
Ternary blends containing PHMS ($f_{\mathrm{PHMS}}=0.5$ and $N_{\mathrm{PHMS}}=30$), *m*-PDMS, and *v*-PDMS. (**a**) Compositions for cases I–VII. (**b**) Equilibrium snapshots for cases I–VI. (**c**) The equilibrium characteristic length scale, $\bar{l}$, for cases I–VII.

To further understand the dependence of the equilibrium characteristic length scale on the properties of the sacrificial components, in the remaining series of simulations we consider ternary systems encompassing a second sacrificial component in addition to the reactive *v*-PDMS chains. In the first example of the ternary system considered, we replace a fraction of *v*-PDMS chains in the system with methyl-terminated PDMS (*m*-PDMS) linear chains, which also serve as the sacrificial component [18,20,58]. We assume that *m*-PDMS chains only consist of the beads of type 5 and do not have any reactive beads. In this series of simulations, we keep the fraction of the matrix at $f_{\mathrm{PHMS}}=0.5$ and vary the fractions and degrees of polymerization of each sacrificial component, specifically $f_{v-\mathrm{PDMS}}$ and $N_{v-\mathrm{PDMS}}$ for *v*-PDMS, and $\left(0.5-f_{v-\mathrm{PDMS}}\right)$ and $N_{m-\mathrm{PDMS}}$ for *m*-PDMS. The details of each system are listed in Figure 5a and the corresponding snapshots for each system in equilibrium are shown in Figure 5b. The corresponding equilibrium characteristic length scales of the sacrificial domains $\bar{l}$ (incorporating all the beads comprising *m*-PDMS and *v*-PDMS chains) for each system are compiled in Figure 5c. The effects of the degree of polymerization of the non-reactive component, $N_{m-\mathrm{PDMS}}$, (cases I–III) and the degree of polymerization of the reactive component, $N_{v-\mathrm{PDMS}}\;$, (cases II, IV, and V) are probed. The increase in the average value of $\bar{l}$ with an increase in $N_{v-\mathrm{PDMS}}$ (cases II, IV, and V) is found to be significantly more pronounced than a moderate increase in $\bar{l}$ with an increase in $N_{m-\mathrm{PDMS}}$ (cases I–III). These trends are consistent with the trends observed in the binary systems (Figure 3c and Figure 4c). Further, a direct comparison between the values of $\bar{l}$ in cases III and V indicates that using longer reactive chains and shorter non-reactive sacrificial chains ($N_{v-\mathrm{PDMS}}=100$, $N_{m-\mathrm{PDMS}}=60$, case V) results in significantly more pronounced increase in the characteristic length scale $\bar{l}$ than using longer non-reactive and shorter reactive chains ($N_{v-\mathrm{PDMS}}=60$, $N_{m-\mathrm{PDMS}}=100$, case III).

The cases VI, II, and VII in Figure 5 illustrate the effect of the relative fraction of the non-reactive phase within the sacrificial component while keeping the degrees of polymerization of both sacrificial components the same $\left(N_{v-\mathrm{PDMS}}=N_{m-\mathrm{PDMS}}=60\right)$. Specifically, we chose $f_{v-\mathrm{PDMS}}=0.3\;$, 0.4, and 0.5 in cases VI, II, and VII, respectively. Recall that the total fraction of the sacrificial component is fixed in these simulations ($f_{v-\mathrm{PDMS}}+f_{m-\mathrm{PDMS}}=0.5$), so that the case VII corresponds to the binary system considered above and is provided in Figure 5 for comparison. These results quantify the decrease in the characteristic length scale $\bar{l}$ with a relative increase in the fraction of the reactive sacrificial component, $f_{v-\mathrm{PDMS}}$.

**Figure 6:**
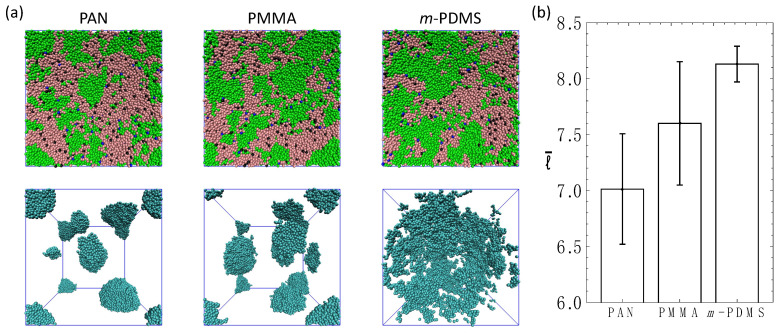
Ternary systems with $f_{v-\mathrm{PDMS}}=0.4$, $f_{\mathrm{PHMS}}=0.5$, $N_{\mathrm{PHMS}}=30$, $N_{v-\mathrm{PDMS}}=N_{X}=60$, where X= PAN, PMMA, *m*-PDMS. (**a**) Simulation snapshots upon equilibration. All beads are shown in the top row (beads of both sacrificial components are in green); and only beads of the non-reactive component (X) are shown in the bottom row. (**b**) Equilibrium characteristic length scale, $\bar{l}$.

Finally, we probe the effect of the chemical nature of the non-reactive sacrificial component on the phase-separated morphology and the characteristic length scale within the system. In the final series of simulations, PAN and PMMA linear chains are used as non-reactive sacrificial components in addition to the *v*-PDMS reactive chains. In effect, these series of simulations correspond to the case II described in Figure 5, but with *m*-PDMS chains replaced with PAN or PMMA chains with the same number of beads. The repulsion parameters $a_{ij}$ used between all the moieties are provided in Table S4 of Supplementary Materials. Note that PAN has stronger repulsion with both PHMS and *v*-PDMS compared to that of PMMA. Due to the miscibility and non-reactivity, *m*-PDMS chains mix and spread within the simulation box along with *v*-PDMS chains (bottom row in Figure 6a, third column). In contrast, due to relatively low affinity between PAN (and PMMA) with both PHMS or *v*-PDMS, we observe that PAN and PMMA chains aggregate and form isolated approximately spherical clusters dispersed in the blends (Figure 6a, bottom row, first two columns). These results show that increasing the repulsion between the non-reactive sacrificial component and matrix (or, similarly, between the non-reactive sacrificial component and reactive sacrificial component) can lead to the pronounced decrease in the characteristic length scale $\bar{l}$ (Figure 6b). Note that $\bar{l}$ in Figure 6b is calculated based on the RDFs of all the sacrificial beads (Figure S5). To summarize, our results point out that introducing non-reactive sacrificial polymer chains could provide a facile method to tailor the morphology of the pores.

## 4. Conclusions

We utilized modified the Segmental Repulsive Potential (mSRP) Dissipative Particle Dynamics (DPD) approach to simulate the phase separation coupled with hydrosilylation in binary and ternary blends. Our approach introduces the first approach to capture hydrosilylation reaction along with the phase separation of preceramic precursors on the mesoscale. Our results shows that the phase separation between polyhydromethylsiloxane (PHMS) and the vinyl-terminated polydimethylsiloxane (*v*-PDMS) is arrested due to the hydrosilylation reaction. We characterized the dynamics of binary PHMS/*v*-PDMS blends undergoing hydrosilylation and ternary blends in which a fraction of the reactive sacrificial component (*v*-PDMS) is replaced with the non-reactive sacrificial component. As the sacrificial non-reactive component in these ternary blends, we chose methyl-terminated PDMS (*m*-PDMS), polyacrylonitrile (PAN), and poly(methyl methacrylate) (PMMA). Our results clearly demonstrate that the morphology of the sacrificial domains in the phase-separated network can be tailored by tuning the composition, chemical nature, and the degree of polymerization of the sacrificial component. Our results show a decrease in the characteristic length scale of the sacrificial domains with either an increase in the fraction of *v*-PDMS chains or a decrease of the degree of polymerization of these chains. We also show that an addition of a non-reactive sacrificial component introduces facile means to control the morphology by varying the fraction, degree of polymerization, or the chemical nature of this component. In particular, a decrease in affinity between the non-reactive sacrificial component and the preceramic precursors results in a decrease in the characteristic length scale of the sacrificial phase in ternary blends. 

## Data Availability

The data presented in this study are available on request from the corresponding author.

## Supplementary Materials

The following supporting information can be downloaded at: https://www.mdpi.com/article/10.3390/nano12183117/s1, Table S1: Solubility parameters and molar volumes of chosen monomers; Table S2: The group molar attraction parameters; Table S3: Flory-Huggins interaction parameters $\chi_{ij}$ (T = 25 °C); Table S4: Repulsion parameters $\alpha_{ij}$ between all the types of beads; Figure S1: Phase separation in the non-reactive blend with $f_{v-\mathrm{PDMS}}=0.5$ and $N_{v-\mathrm{PDMS}}=100$ (no hydrosilylation reactions); Figure S2: Comparison between characteristic length scales defined by l and $l_{min}$ in the system shown in Figure 2 of the main text ($f_{v-\mathrm{PDMS}}=0.5$ and $N_{v-\mathrm{PDMS}}=100$); Figure S3: Effects of reaction probability, $P_{c}$; Figure S4: Snapshots of sacrificial domains in the blends with $N_{v-\mathrm{PDMS}}=60$ and various $f_{v-\mathrm{PDMS}}$; Figure S5: Shifted RDFs in ternary systems corresponding to Figure 6 at equilibrium. References [52,57,68,69,70,71,72,73,74] are cited in the supplementary materials.
