# Supplementary material for: Mesoscale Modeling of Phase Separation Controlled by Hydrosilylation in Polyhydromethylsiloxane (PHMS)-Containing Blends
Source: Nanomaterials (Basel). 2022 Sep 8;12(18):3117. doi: 10.3390/nano12183117 (PMC9502167; doi:10.3390/nano12183117)
Supplement: Supplementary file 1 [file nanomaterials-12-03117-s001.zip › nanomaterials-1845277-supplementary.pdf]

SUPPLEMENTARY MATERIALS

Mesoscale Modeling of Phase Separation Controlled by Hydrosilylation in Polyhydromethylsiloxane (PHMS)-Containing Blends

Yao Xiong [1], Chandan K. Choudhury [1,2], Vaibhav Palkar [1], Raleigh Wunderlich [1,3], Rajendra K. Bordia [1] and Olga Kuksenok [1,*]

[1] Department of Materials Science and Engineering, Clemson University, Clemson, SC 29634, USA;

[2] Prescience Insilico Pvt. Ltd., Bengaluru 560037, Karnataka, India

[3] Georgia Institute of Technology, Atlanta, GA 30332, USA

* Correspondence: okuksen@clemson.edu

Supplementary Tables

Table S1. Solubility parameters and molar volume of selected monomers

POLYMER	$\delta\ (\mathrm{cal/cm^3})^{1/2}$	$V_m\ (\mathrm{mL/mol})^\dagger$
Poly(dimethylsiloxane) (PDMS)	7.3[1]	76.3–76.7[4] (76.7)
Polyacrylonitrile (PAN)	12.75[2]	44.8[4]
Poly(methyl methacrylate) (PMMA)	9.45[3]	85.6–89.3[4] (87.1)
Poly(hydromethylsiloxane) (PHMS)	7.46‡	59.8††

†Values in the brackets are used for calculations in this study.

‡This value is calculated using the group molar attraction constants provided in Table S2. The density of PHMS is $\rho_{\mathrm{PHMS}} = 1.006\ \mathrm{g/mL}$ (CAS Number 63148-57-2, Sigma-Aldrich, USA) and the molecular weight of repeat unit is $M_{\mathrm{PHMS}} = 60.127\ \mathrm{g/mol}$.

††This value is calculated using $M_{\mathrm{PHMS}}/\rho_{\mathrm{PHMS}}$.

Table S2. The group molar attraction parameters

GROUP	$F\ (\mathrm{cal \cdot cm^3})^{1/2}/\mathrm{mol}$
-O-	70[3]
-H	80–100 (80)[3]
$-CH_3$	214[3]
$-CH_2-$	133[3]
-CH<	28[3]
>C<	–93[3]
Si	82-102 (82)[5]

Table S3. Flory-Huggins interaction parameters χ_{ij} (T=25 ºC)

χ_{ij}	PDMS	PAN	PMMA	PHMS
**PDMS**	0	3.046	0.639	0.343
**PAN**		0	1.212	2.470
**PMMA**			0	0.491
**PHMS**				0

Table S4. Repulsion parameters a_{ij} (T=25 ºC)

a_{ij} (k_BT/r_c)	PDMS	PAN	PMMA	PHMS
**PDMS**	78.00	87.96*	80.09**	79.12+
**PAN**		78.00	81.96	86.08++
**PMMA**			78.00	79.61§
**PHMS**				78.00

*a_{ij} between bead type 1, 3, or 5 with beads of PAN chain

**a_{ij} between bead type 1, 3, or 5 with beads of PMMA chain

+a_{ij} between bead type 1, 3, or 5 with bead type 2, 4, or 6 (end beads of PHMS chain)

++a_{ij} between beads of PAN chain with bead type 2, 4, or 6 (end beads of PHMS chain)

§a_{ij} between beads of PMMA chain with bead type 2, 4, or 6 (end beads of PHMS chain)

Supplementary Figures

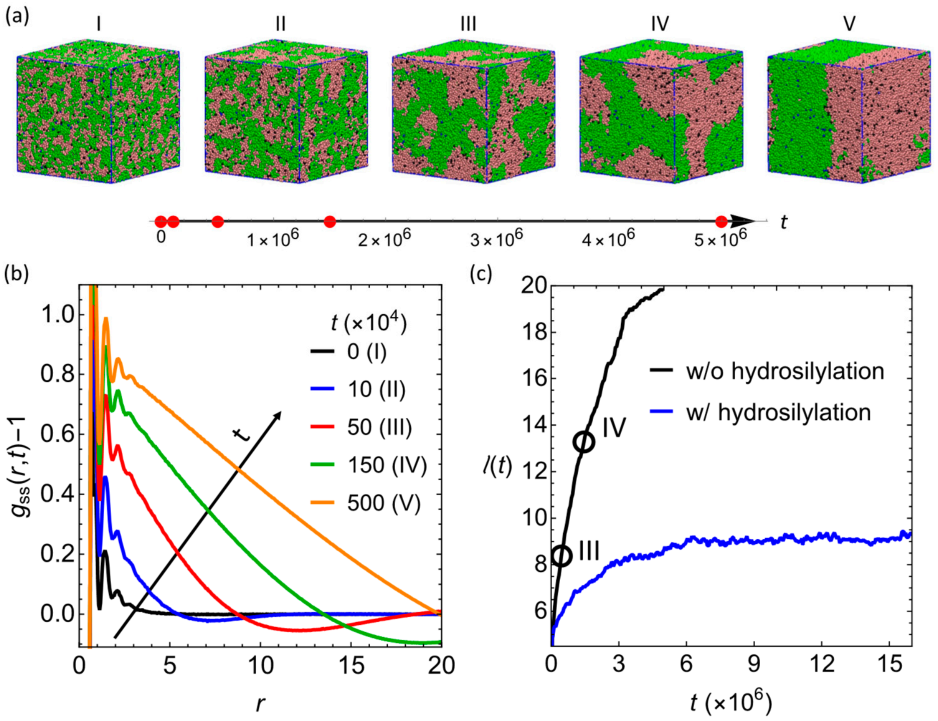

Figure S1. Phase separation in the non-reactive blend with $f_{v\text{-PDMS}} = 0.5$ and $N_{v\text{-PDMS}} = 100$ (no hydrosilylation reactions). (a) Snapshots (I-V) of the blend at (from the left to the right) $t = 0$, 1×10^5, 5×10^5, 1.5×10^6, and 5×10^6. **(b)** The $g_{ss}(r,t) - 1$ at various times as listed in the legend. **(c)** The time evolution of the characteristic length scale, $l(t)$, in the absence of the hydrosilylation reaction (in black) and with hydrosilylation reaction (in blue). The time instances corresponding to the snapshots in (a) are marked by the open circles.

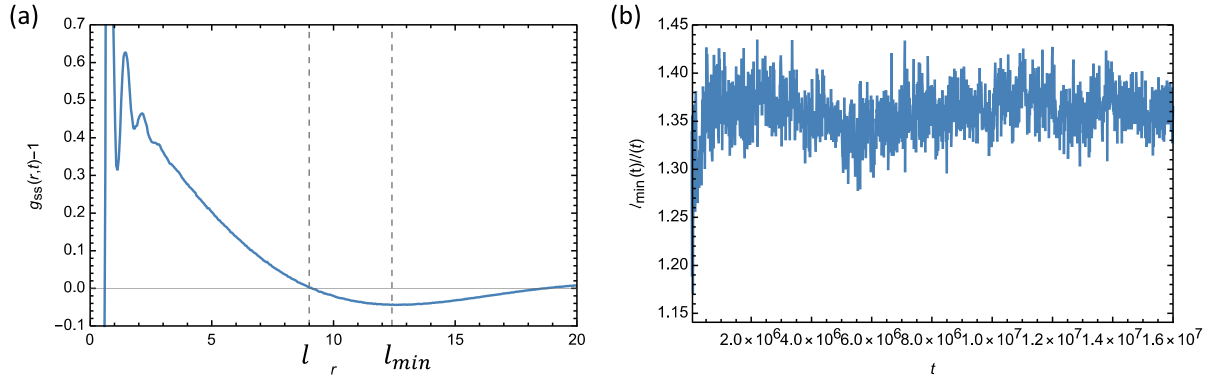

Figure S2. Comparison between characteristic length scales defined by l and l_{min} in the system shown in Figure 2 of the main text ($f_{v-\mathrm{PDMS}} = 0.5$ and $N_{v-\mathrm{PDMS}} = 100$). (a) Shifted radial distribution function, $g_{ss}(r,t) - 1$, for the case considered in Figure 2 at $t = 1.2 \times 10^7$. Characteristic sizes given by the first zero crossing of $g_{ss}(r,t) - 1$ and by the minimum of $g_{ss}(r,t)$ at this time instant are marked by l and l_{min} , respectively. **(b)** Time evolution of the ratio $l_{min}(t)/l(t)$.

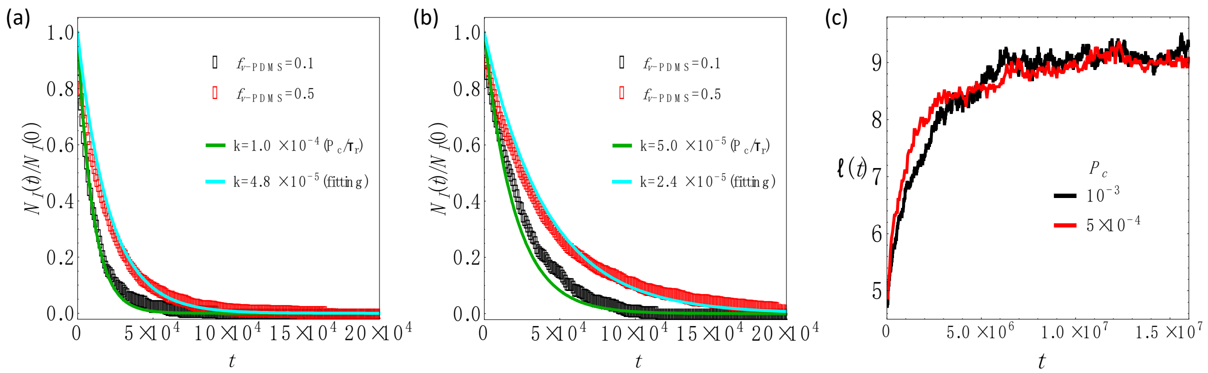

Figure S3. Effects of reaction probability, P_c. (a-b) Dynamics of the number of vinyl groups remaining reactive in the system normalized by the initial value of these reactive groups, $N_I(t)/N_I(0)$, for systems with $N_{v-\mathrm{PDMS}} = 100$ and $f_{v-\mathrm{PDMS}} = 0.1$ (black symbols) and $f_{v-\mathrm{PDMS}} = 0.5$ (red symbols), $P_c = 10^{-3}$ in (a) and $P_c = 5 \times 10^{-4}$ in (b). **(c)** Time evolution of characteristic length scale $l(t)$ in systems with $f_{v-\mathrm{PDMS}} = 0.5$ and $N_{v-\mathrm{PDMS}} = 100$ at two probabilities as listed in the legend.

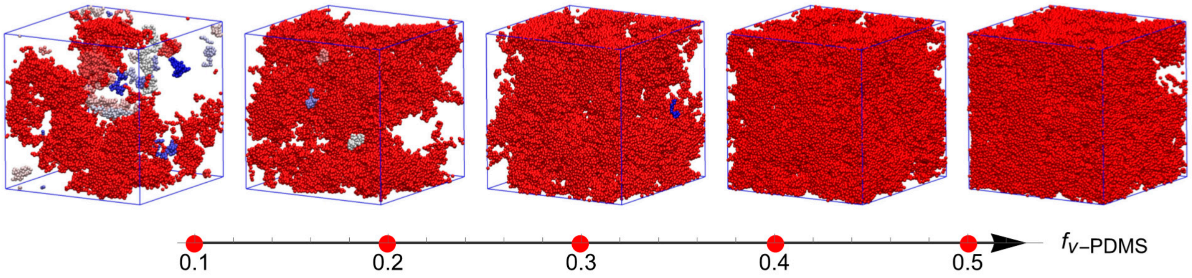

Figure S4. Snapshots of the sacrificial domains in the blends for the simulation runs shown in Figure 4a of the main text ($N_{v-\mathrm{PDMS}} = 60$). The values of $f_{v-\mathrm{PDMS}}$ from the left to the right are 0.1, 0.2, 0.3, 0.4, and 0.5, respectively. Two beads are considered to belong to the same cluster if the distance between the centers of these beads is smaller than r_c. Different colors are used to mark each individual cluster. Visual Molecular Dynamics (VMD) software [6] is used to identify clusters for a given trajectory. The total number of clusters identified using this criterium for the snapshots from the left to the right are 18, 5, 3, 1, and 1, respectively. Hence, a majority of sacrificial beads forms a largest interconnected cluster for cases with lower $f_{v-\mathrm{PDMS}}$ (see clusters marked in red in three first images from the left to the right), and all the sacrificial beads belong to the single cluster for higher values of $f_{v-\mathrm{PDMS}}$ (two images on the right corresponding to $f_{v-\mathrm{PDMS}} = 0.4$ and $f_{v-\mathrm{PDMS}} = 0.5$, respectively).

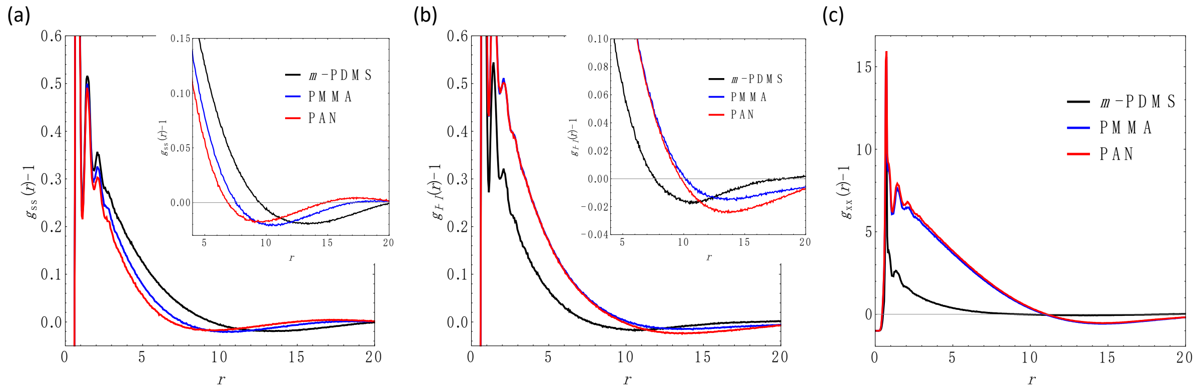

Figure S5. Shifted RDFs in ternary systems corresponding to Figure 6 at equilibrium. (a) Shifted partial RDFs of all sacrificial beads, $g_{ss}(r) - 1$. **(b)** Shifted partial RDFs of *v*-PDMS chains (beads of types 1, 3, and 5), $g_{I-I}(r) - 1$. **(c)** Shifted partial RDFs of non-reactive component, $g_{xx}(r) - 1$.

REFERENCES

[1] J. N. Lee, C. Park and G. M. Whitesides, *Anal. Chem.*, 2003, **75**, 6544-6554.

[2] J. E. Mark, *Physical Properties of Polymers Handbook*, Springer, New York, 2007.

[3] L. H. Sperling, *Introduction to Physical Polymer Science*, Wiley, Hoboken, 2015.

[4] Polymer database, http://www.polymerdatabase.com, (accessed July 2022)

PDMS: https://polymerdatabase.com/polymers/Polydimethylsiloxane.html, (accessed July 2022)

PAN: https://polymerdatabase.com/polymers/polyacrylonitrile.html, (accessed July 2022)

PMMA: https://polymerdatabase.com/polymers/polymethylmethacrylate.html, (accessed July 2022)

[5] H. Watanabe and T. Miyauchi, *J. Chem. Eng. Jpn.*, 1973, **6**, 109-114.

[6] W. Humphrey, A. Dalke and K. Schulten, *J. Mol. Graphics Modell.*, 1996, **14**, 33-38.
